# Corrosion of Passive Aluminum Anodes in a Chloroaluminate Deep Eutectic Solvent for Secondary Batteries: The Bad, the Good, and the Ugly

**DOI:** 10.1021/acsami.2c16153

**Published:** 2022-12-27

**Authors:** David Moser, Philipp Materna, Anna Stark, Judith Lammer, Attila Csík, Jasmin M. Abdou, Raphael Dorner, Martin Sterrer, Walter Goessler, Gerald Kothleitner, Bernhard Gollas

**Affiliations:** † Institute of Electron Microscopy and Nanoanalysis, Graz University of Technology, Steyrergasse 17, 8010Graz, Austria; ‡ Institute for Chemistry and Technology of Materials, Graz University of Technology, Stremayrgasse 9/II, 8010Graz, Austria; § Graz Centre for Electron Microscopy, Steyrergasse 17, 8010Graz, Austria; ∥ Institute for Nuclear Research, Bem ter 18/c, 4026Debrecen, Hungary; ⊥ Institute of Physics, University of Graz, Universitätsplatz 5, 8010Graz, Austria; # Institute of Chemistry, University of Graz, Universitätsplatz 1, 8010Graz, Austria

**Keywords:** aluminum anode, native oxide, aluminum−sulfur battery, chloroaluminate electrolyte, corrosion, trace metal impurities, pitting, local current distribution

## Abstract

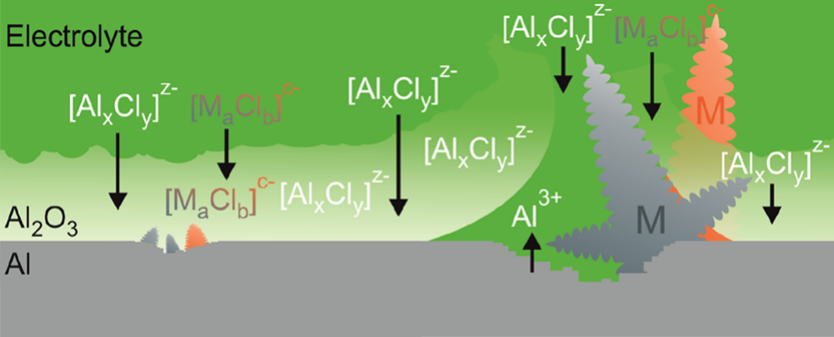

The passivity of aluminum is detrimental to its performance as an anode in batteries. Soaking of native oxide-covered aluminum in a chloroaluminate deep eutectic solvent gradually activates the electrode surface, which is reflected in a continuously decreasing open circuit potential. The underlying processes were studied by analyzing the 3 to 7 nm thick layer of native oxide after increasing periods of soaking with secondary neutral mass spectrometry, X-ray photoelectron spectroscopy, and energy-dispersive spectroscopy in a transmission electron microscope. They consistently show permeation of electrolyte species into the layer associated with gradual swelling. After extended periods of soaking at open circuit potentials, local deposits of a range of foreign metals have been found in scanning electron microscopy images of the electrode surface. The pitting corrosion is caused by trace metal ion impurities present in the electrolyte and results in highly nonuniform current density distribution during discharge/charge cycling of battery cells as shown by local deposits of aluminum. The processes during soaking at open circuit potentials have been monitored by electrochemical impedance spectroscopy and could be analyzed by fitting an equivalent circuit model for pitting corrosion.

## 1 Introduction

Aluminum is a technologically very important metal in numberless applications, predominantly as a light-weight construction material. Despite its negative standard potential, it is protected from corrosion in many environments by its native oxide layer that spontaneously forms under ambient conditions. This passivating layer can easily be reinforced by anodizing processes and acts as a kinetic barrier that prevents the underlying thermodynamically reactive metal from further oxidation. However, in media that contain aggressive anions such as chloride, the passive oxide film can be attacked, leading to a local breakdown and corrosion. In order to find ways to prevent or mitigate this process in aqueous media, it has been studied extensively over the last decades and the findings have been summarized.^1^ A range of methods including X-ray photoelectron spectroscopy (XPS),^2−5^ electrochemical impedance spectroscopy (EIS),^6,7^ X-ray absorption near edge structure,^8^ and secondary ion mass spectrometry^9^ have been used to characterize the interaction between the oxide film and chloride ions in aqueous solution. They all show that chloride ions permeate into the oxide layer before pitting occurs. Bucko et al. have studied the stability of two commercial aluminum alloys in a deep eutectic solvent consisting of urea and choline chloride and reported no significant corrosion.^10^

More recently, a possible application of aluminum has become the focus of attention, where its passive state is detrimental. Rechargeable batteries play a key role in realizing an uninterrupted renewable energy supply in a future more sustainable economy. Currently, the market is dominated by lithium-based battery systems, but this technology faces challenges of safety, limited resources, and high costs.^11^ This drives tremendous efforts to replace lithium in secondary batteries by safer chemistry, based on more abundant and significantly cheaper materials. Aluminum fulfills these criteria because it is the most abundant metal in the earth’s crust and, hence, a cheap resource with a high recycling rate. Its theoretical value of volumetric capacity of 8040 mAh·cm^–3^ is superior, and its specific capacity of 2980 mAh·g^–1^ is similar to that of lithium metal.^12−14^ In combination with either insertion cathodes made from graphitic carbon^15^ or conversion cathodes based on, e.g., sulfur^16,17^ or oxygen,^18^ aluminum anodes could yield a highly promising battery chemistry, provided a suitable electrolyte can be found.

Most studies on room-temperature secondary aluminum batteries have been performed with chloroaluminate ionic liquids (ILs).^12,19^ Due to the high cost of these electrolytes, however, alternatives have been looked for and recently chloroaluminate deep eutectic solvents (DESs) based on mixtures of AlCl_3_ and amides such as urea have been suggested.^15,20,21^ Such DESs are particularly interesting because of their low cost and nontoxicity.^22,23^ In order to allow reversible dissolution and deposition of aluminum (eq 1) during discharging and charging, both ILs and DESs have to be Lewis acidic; i.e., the molar fraction of AlCl_3_ in the mixtures must be larger than 0.5. In this case, the dominating electroactive anion in both types of electrolytes is [Al_2_Cl_7_]^−^.^21,24,25^ In DESs, cationic aluminum species can additionally contribute to dissolution and deposition (eq 2), which then adds up to the reaction in eq 3.^26^
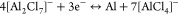
1
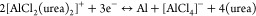
2
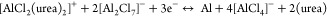
3

The native oxide layer present on the surface of aluminum behaves as an n-type semiconductor^7^ and is known to limit its electrochemical activity.^14^ Chen et al. nevertheless suggested that the native oxide layer on aluminum foil should be advantageous for secondary Al batteries.^27^ The insulating alumina could potentially restrict dendrite growth by confining noncompact deposits under the oxide layer, preventing disintegration of the anode foil during extensive discharge/charge cycling. Most reports, however, describe the passive nature of the oxide-covered Al anode as detrimental to a voltage-efficient battery.^28−30^ If the battery is assembled in the charged state (e.g., with a sulfur cathode), the passivating native oxide layer on the aluminum anode poses a significant challenge to an efficient battery discharge/charge performance. The oxide layer shields the aluminum from the electrolyte and causes considerable overpotentials during discharging/charging (dissolution/deposition).^12^

It has been reported, however, that soaking the aluminum anode in suitable nonaqueous electrolytes at open circuit potential (ocp) has a positive effect on its electrochemical performance,^24,28−32^ whereby the disruption of the oxide layer and degree of activation was shown to increase with time. Wang et al. showed the necessity of immersing the electrode in a corrosive, chloride-containing IL electrolyte in order to produce an electrochemically active aluminum surface for efficient dissolution/deposition of aluminum.^29^ Wu et al. showed an improvement of the electrochemical performance of the aluminum anode upon pre-treatment with AlCl_3_/butyl-methyl-imidazolium chloride.^31^ Lee et al. investigated different IL compositions and suggested that electroactive regions are generated by local dissolution of the oxide layer and redeposition in the immediate vicinity.^24^ Shvartsev et al. have shown that the oxide film on aluminum reacts with oligo-fluoro-hydrogenate anions in EMIm(HF)_2.3_F electrolyte and forms an Al–O–F layer that allows efficient Al electrochemical dissolution.^28^ It seems to be accepted that the interaction of chloroaluminate-containing electrolyte with the native oxide-covered aluminum anode at ocp results in some form of corrosion,^24,29^ although it is not clear what oxidizing species could be responsible. In contrast to oligo-fluoro-hydrogenate, there are no protons available in chloroaluminate ILs and DESs. In addition, the concentration of free chloride ions in these Lewis acidic electrolytes is probably negligible because the equilibrium constant of the autosolvolysis reaction of [AlCl_4_]^−^ (eq 4) in the similar mixture of AlCl_3_ and 1-ethyl-3-methylimidazolium chloride is about 10^–17^.^33^
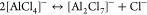
4

Furthermore, it is not known whether the oxide layer chemically reacts with chloroaluminates. Unfortunately, some of the most detailed studies have been performed on samples with artificially thickened oxide layers.^28,34^ Long et al. claimed the dissolution of the oxide layer in a chloroaluminate IL while indicating the simultaneous formation of a solid electrolyte interphase.^34^ What is more, the surface characterization of aluminum is hampered by its high reactivity toward oxygen and water even at the low levels found in inert gas glove boxes.^35−37^ Improper sample preparation, such as cleaning of aluminum with water or ethanol after immersion in chloroaluminate electrolytes, causes hydrolysis of AlCl_3_ and chloroaluminates into Al(OH)_6_^3–^ and Cl^–^, and the resulting residues can lead to artifacts and misinterpretation.

Despite the aforementioned reports in the literature on the beneficial effects of pre-immersing Al electrodes into IL or DES electrolytes for secondary batteries before cycling, no satisfactory explanation of such effects has been given. In this publication, a detailed study is presented of the processes occurring on the native oxide-covered aluminum upon immersion in a 1.5:1 molar ratio deep eutectic mixture of AlCl_3_ and urea (Uralumina 150). In particular, we will show that chlorometalate species slowly permeate the oxide layer. Impurities of foreign metal ions are chemically reduced on the aluminum surface resulting in local galvanic corrosion and the formation of pits. Progressive pitting causes underetching and peeling of the oxide layer leading to local activation of the aluminum surface that increases with soaking time.

## 2 Experimental Section

### 2.1 Chemicals and Materials

The Uralumina 150 electrolyte consisted of AlCl_3_ (abcr, anhydrous, 99.99%) and urea (Sigma-Aldrich, dry, ≥99.5%) in a molar ratio of 1.5:1. All electrolyte preparation steps were carried out under inert gas in an Ar-filled glove box (GS Alpha Line, Germany, H_2_O < 1 ppm, O_2_ < 1 ppm). The urea was grinded with a glass rod to crush agglomerates before adding it to the AlCl_3_. Urea was added stepwise in small portions and under vigorous mixing with a glass rod, allowing the mixture to cool after each step, before adding the next portion. After the addition of urea was completed, the colorless suspension was stirred for 12 h on a magnetic stirrer. The suspension was filtered through a syringe filter (Macherey-Nagel, Chromafil GF, 1 μm) leading to a clear colorless liquid. Acetlumina 150 was prepared analogously by mixing AlCl_3_ and acetamide (Sigma-Aldrich, >99.0%,). The IL 1.5:1 mixture of AlCl_3_ and 1-ethyl-3-methylimidazolium chloride (≥95%) was purchased from Sigma-Aldrich.

### 2.2 Inductively Coupled Plasma Mass Spectrometry

Trace metal impurities were determined with an Agilent 7700x inductively coupled plasma mass spectrometer after simple 1000-fold dilution of the electrolyte. After a semiquantitative element-screening, Fe, Ni, Zn, Y, Cd, In, Gd, Pb, and U were quantified with external calibrations in the range from 0.5 to 50 μg/L for Fe and Zn and from 0.05 to 5 μg/L for all other elements. To reduce polyatomic interferences, Fe, Ni, Zn, Y, Cd, In, and Gd were analyzed in the collision mode using 4.5 mL/min He. For quality control, the certified reference material NIST 1640a (trace element in water) was analyzed together with the samples. Good agreement with the certified concentrations has been obtained.

### 2.3 Aluminum Electrodes

Al disc working and counter electrodes used in the Swagelok cells had a diameter of 6 mm (99.999% purity, Advent Research Materials) embedded in a 10.4 mm diameter poly(tetrafluoroethylene) shroud. The Al disc working electrode used in the three-electrode glass cell for ocp monitoring and EIS had a diameter of 3 mm (99.999% purity, Advent Research Materials) embedded in a 10 mm diameter Teflon shroud. The disc electrodes were polished to a mirror finish with SiC grinding paper (Struers, mesh size #1200, #2400, and #4000) followed by polishing with 3 μm diamond suspension (Struers), cleaned with soap, and rinsed with distilled water. The Al electrodes were then sonicated in an ultrasound bath (Emag Germany emmi-4) with ethanol (ROTH ethanol 96%) for 5 minutes, followed by a rinse with deionized water and drying in an oven for 48 h at 60 °C before transferring them into the glove box. The Al reference electrode was an Al wire with a 1 mm diameter (99.999% purity, Advent Research Materials), which was briefly abraded with emery paper before assembly.

Thin film (100 nm) Al layers (8 × 8 mm^2^) were deposited by physical vapor deposition (PVD) onto standard Si wafers (10 × 10 × 1 mm^3^) under an aluminum mask (9 × 9 mm^2^ openings) in a vacuum chamber (10^–5^ bar) connected to a glove box. They were then exposed to ambient air for 2 days to allow formation of the native oxide layer before transferring them back into the glove box, where they were immersed in the Uralumina 150 electrolyte for certain periods of time. Afterward, the electrolyte was rinsed off with anhydrous glyme (99.5%, Sigma-Aldrich) and left to dry in the glove box. The dried thin film samples were then either coated by PVD in the glove box with an Au layer (115 nm thickness) before transferring them to either the secondary neutral mass spectrometer or the electron microscopes or locked into a gas-tight sample container for transfer to the X-ray photoelectron spectrometer.

### 2.4 Electrochemistry

Battery cells made from perfluoroalkoxy alkane Swagelok tee fittings were used for the three-electrode configurations in galvanostatic cycling experiments. The Swagelok cells equipped with working and counter electrodes were filled with Uralumina 150 through the opening for the reference electrode, fitted with the reference electrode, closed tightly, and connected to a Basytec CTS LAB XL (Basytec GmbH, Asselfingen, Germany) for galvanostatic discharge/charge cycling. The ocp was monitored in the Swagelok cells or in a conventional three-electrode glass cell in the argon-filled glove box with a Metrohm Autolab B.V. PGSTAT302N controlled by Metrohm NOVA 1.11 software. EIS was carried out also in the glass cell with a Zahner IM6 workstation. Spectra were analyzed by fitting the parameters of the chosen equivalent circuit to the measured data in ZView4 (Scribner Associates, Inc.).

### 2.5 Secondary Neutral Mass Spectrometry

Elemental depth profiles were measured with secondary neutral mass spectrometry (SNMS, type INA-X, SPECS GmbH) in the direct bombardment mode using Ar^+^ ions with a plasma pressure of 1.5 × 10^–3^ mbar and with a current density of ≈1 mA·cm^–2^. The samples were sputtered with 350 V at 100 kHz frequency and 80% duty cycle. Post-ionized neutral particles are directed into a quadruple mass spectrometer Balzers QMA 410 (up to 340 AMU) by electrostatic lenses and a broad pass energy analyzer. A round-shaped erosion area was confined to a circle of 2 mm in diameter by means of a Ta mask. The lateral homogeneity of the ion bombardment was checked with a profilometer (Ambios XP I) to analyze the depth of the sputtered crater. The sputtering time was converted to depth by calibration of sputtering rates with results obtained from the profilometer measurements. After fabrication of the thin film samples by physical vapor deposition (PVD) on Si wafers, they were exposed to ambient air for 2 days in order to grow the native oxide. They were then immersed for increasing periods of time in Uralumina 150 in the glove box and subsequently rinsed with anhydrous glyme and dried. Before removing them from the glove box for transfer to the SNMS, they were coated with a 100 nm thick Au film in the PVD facility integrated in the same glove box to protect them from moisture.

### 2.6 X-ray Photoelectron Spectroscopy

XPS was carried out in a custom-built ultrahigh-vacuum (UHV) chamber system (base pressure 2 × 10^–10^ mbar) equipped with a load-lock for fast sample transfer, a sputter gun, and a dual-anode X-ray gun (SPECS XR50) and a hemispherical electron analyzer (SPECS Phoibos 150). X-ray photoelectron spectra were acquired with Al Kα excitation at normal photoelectron emission (for specific measurements, e.g., film thickness determination, the photoelectron emission angle was varied). The samples were mounted on a flag-style sample holder and introduced into the vacuum chamber as received. Spectra were taken from the as-received samples and from samples that had been sputtered for various periods with Ar ions (typical sputter conditions: 1 × 10^–5^ mbar Ar, 3–10 μA sputter current). After soaking in Uralumina 150 in the glove box, the thin film Al on Si samples could not be protected with a sufficiently thick Au layer because the sputtering rate in the spectrometer is too low. Instead, the rinsed and dried samples were locked in the glove box in a gas-tight sample chamber that could be flanged to the UHV chamber of the spectrometer. This turned out to effectively prevent degradation of the samples during transfer.

### 2.7 Electron Microscopy

A Sigma 300 VP (Zeiss, Germany) scanning electron microscope equipped with an Oxford SDD 30 EDS detector (Oxford Instruments, United Kingdom) was used. Transmission electron microscopy (TEM) lamellae were prepared by an FEI Nova 200 NanoLab (FEI, USA), a dual beam focused ion beam (FIB)/SEM microscope, and mounted at an Omniprobe Cu grid. Measurements were performed with an FEI Titan^3^ G2 60-300 (FEI, USA) equipped with a CS-probe corrector, a Super-X EDS detector (FEI, NL), and a Gatan Image Filter Quantum ERS (Gatan, USA) as well as a K2 direct electron detection camera (Gatan, USA). All TEM measurements were carried out at 300 kV acceleration voltage and in monochromated STEM mode. Elemental maps and line scans were obtained using GMS 3 (Gatan, USA). The HAADF SE image of the impurity deposit and corresponding TEM-EDS maps (Figure S6b–h) were acquired and processed using Velox Version 2 (Thermo Fischer Scientific, USA). As for SNMS and XPS, it was necessary also for TEM to use thin film samples because the surface of mechanically polished Al is too rough.

## 3 Results and Discussion

### 3.1 Passivity of the Aluminum Anode

**Figure 1:**
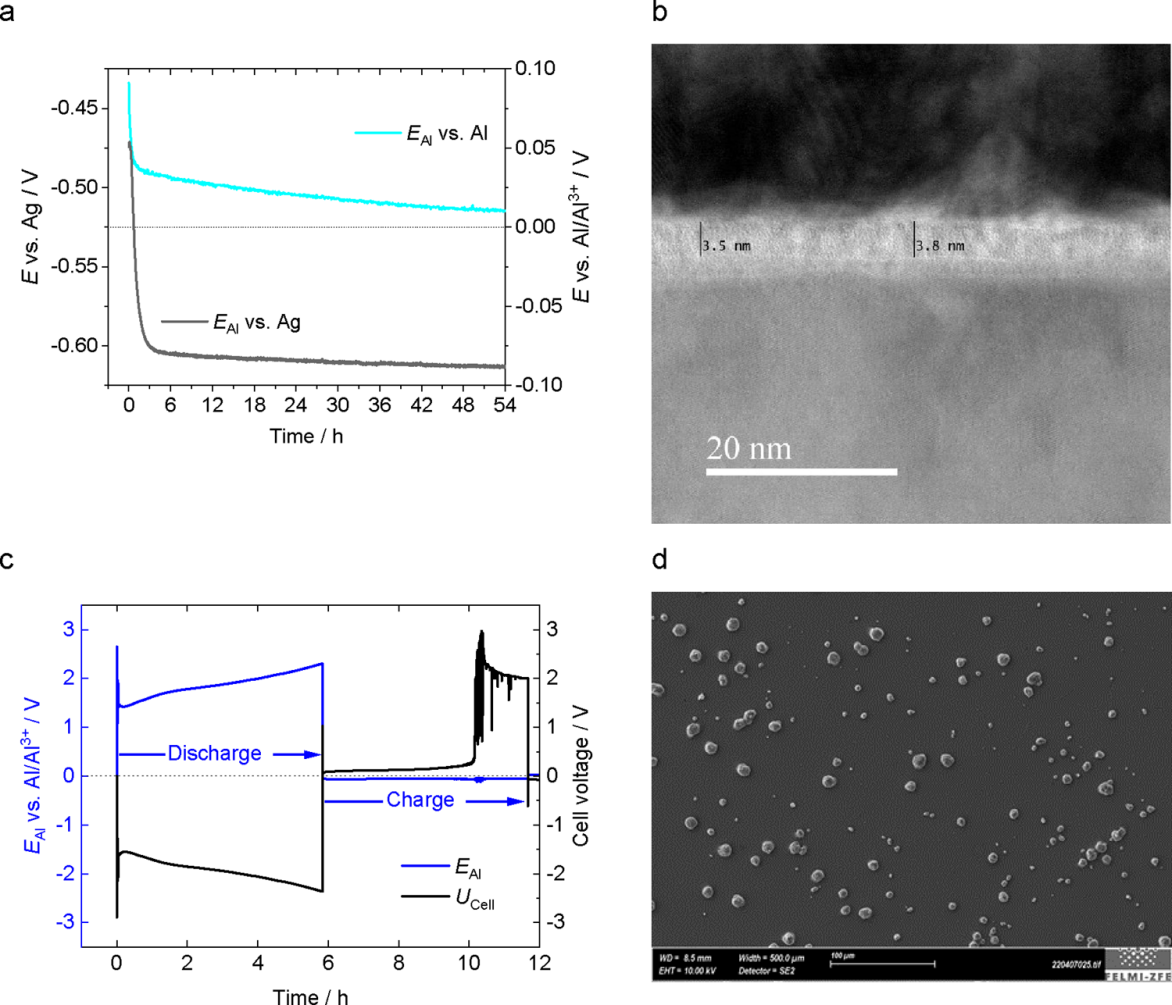
(a) Progression of the open circuit potential of aluminum electrodes covered with native oxide soaking in Uralumina 150. (b) TEM cross-section of the native oxide layer on an Al disc electrode. (c) Potential *E* of the negative Al electrode and cell voltage *U* during galvanostatic discharge/charge cycle (0.067 mA·cm^–2^, 1 mA·h·cm^–2^) in a symmetrical Swagelok-type battery cell with Uralumina 150 electrolyte. (d) SEM image of an Al electrode after 1 h of galvanostatic Al deposition (0.217 mA/cm^2^) in a symmetrical Swagelok type battery cell with Uralumina 150.

A typical progression of the ocp in a three-electrode symmetric Swagelok cell equipped with an Al wire reference electrode is shown in Figure 1a. It starts at very positive potentials after immersion of the aluminum electrode in the chloroaluminate deep eutectic electrolyte. Potentials above +0.6 V vs Al wire immediately after electrode immersion can be measured in a three-electrode glass cell, where the measurement can be started directly with electrode insertion. The high initial ocp is likely caused by the passive and largely amorphous native oxide layer, which usually has a thickness between 3 and 4 nm as observed in cross sections by TEM (Figure 1b). Over the course of several hours, the potential drops to values of a few mV above 0 V. A similar behavior has been observed for artificially thickened Al oxide immersed in EMIm(HF)_2.3_F electrolyte^28^ and explained with the oxide-covered aluminum losing its passivity by some kind of surface activation. The difference here is that the observed potential change occurs on a considerably longer timescale. The fact that the potential does not converge to 0 V vs Al indicates a mixed potential probably as the result of a persistent corrosion process (vide infra). This is surprising because, in the oxygen and water-free Uralumina 150 and by contrast to the aforementioned EMIm(HF)_2.3_F, there are no obvious oxidants such as protons available in the deep eutectic electrolyte. In a few measurements, the ocp dropped also slightly below 0 V vs Al wire, which indicates that the Al wire reference electrode might also be affected by these processes. Shvartsev et al. have introduced a reliable reference electrode based on the ferrocene/ferrocenium couple for use in ILs.^37^ In contrast to Al wire, we have found that Ag wire shows a stable potential in Uralumina 150. A corresponding ocp trace vs Ag wire is shown also in Figure 1a. The steady decrease of the ocp indicates a continuous activation of the Al surface. Constant current cycling in a symmetrical Swagelok-type battery cell with Uralumina 150 shows considerable overpotentials at the negative electrode during discharge (Figure 1c) and produces only local deposits of Al statistically distributed over the electrode surface (Figure 1d) during the first charging half-cycle. The same behavior was observed (Figure S1) with the DES Acetlumina 150 and the IL of a 1.5:1 molar mixture of AlCl_3_ and 1-ethyl-3-methylimidazolium chloride. For a better understanding of the behavior of the aluminum electrode in Uralumina 150, the compositional changes of the oxide layer after various periods of soaking in the electrolyte at ocp were studied with a range of methods, which will be described in the following sections.

### 3.2 Electrolyte Permeation of the Native Aluminum Oxide

**Figure 2:**
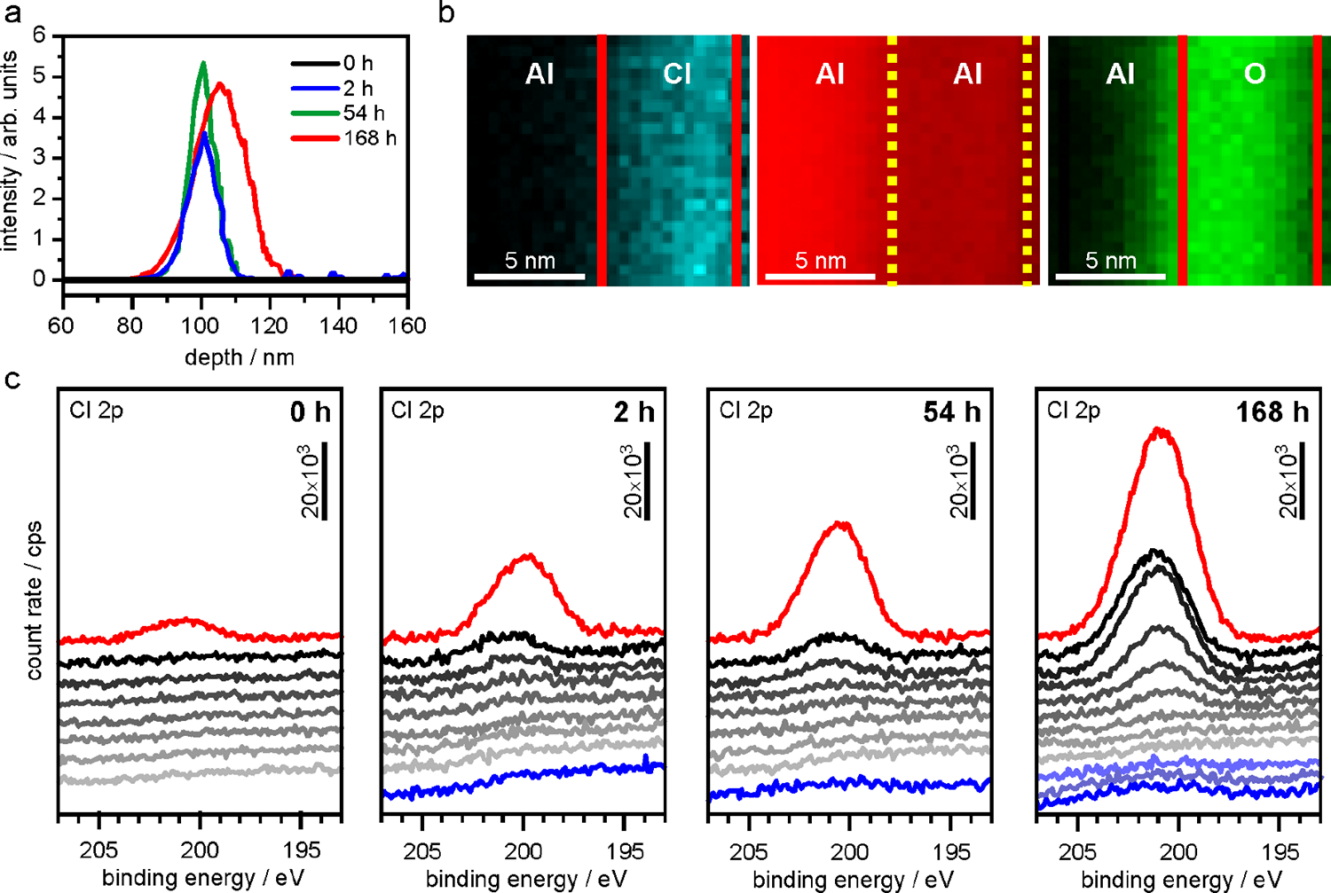
Analysis of native oxide layer. (a) SNMS depth profiles of Cl in native Al oxide after 0, 2, 54, and 168 h of electrolyte immersion. (b) EDS maps of Cl, Al, and O in the Al oxide layer after 168 h of immersion. Dashed yellow lines indicate the presence of EELS Al^3+^ ELNES. (c) XPS sputter profiles of Cl in native Al oxide after 0, 2, 54, and 168 h of immersion (red: as-received sample; black-gray: progressive change in the Al film with an increasing number of sputter cycles. Blue: after almost complete removal of the Al film).

Samples of native oxide-covered aluminum were soaked for increasing periods of time in Uralumina 150 in the glove box. Elemental depth profiles have been recorded by SNMS during plasma sputtering of a circular area of 2 mm diameter with Ar^+^ ions. Since the native oxide layer is only about 5 nm thick, smooth aluminum thin film samples had to be used in order to maximize the depth resolution and produce sharp interfaces. Figure S2 shows the elemental depth profiles of a pristine sample and samples that had been soaked for periods of 2, 54, and 168 h in Uralumina 150. The signal of the native oxide is visible in between the protecting Au and the Al layer. The oxygen peak was analyzed and the full-width at half maximum was calculated in order to determine the layer thickness. We found that the thickness of the layer was 6.5 nm, which is in good agreement with the results obtained from XPS (vide infra). The chlorine signal stretches across that of the oxide layer indicating chlorometallates have permeated the passive barrier. The uptake of chloro-species in the layer depends on the length of the soaking period as shown in Figure 2a. The amount of chlorine and the penetration depth in the oxide layer increased significantly with immersion time.

In order to obtain chemical information also on the bonding state of the elements in the oxide layer, XPS was used. We show the detailed spectra of the Cl 2p (Figure 2c), Al 2p, O 1s, and N 1s regions (Figure S3) for the pristine sample, and samples soaked for 2, 54, and 168 h, respectively. The upmost (red) spectra were obtained from the samples in the as-received state, while the black-gray-blue spectra show the progressive changes with increasing sputter cycles. As-received samples were covered with a layer of weakly bound residual electrolyte and thus exhibited large signals due to Cl, N, and C (not shown), which were removed by short sputtering. Chloride and nitrogen signals remaining after the first sputtering originate from species strongly bound in the surface layer of the electrode after permeation with electrolyte. The oxide layer thickness was determined by comparison of the Al^3+^ and the Al^0^ signal according to the procedure reported by Marcus et al.,^38^ yielding 3.8 nm oxide on the blank sample, which increases with longer immersion time and reaches 6.7 nm after 168 h soaking (Table 1).

**Table 1:** Aluminum Oxide Thickness Measured by XPS after Different Immersion Periods

immersion period/h	oxide thickness before sputtering/Å
0	37.8
2	47.5
54	49.1
168	67.3

The Al 2p and O 1s signals show the typical changes expected for progressive sputtering of the oxide layer. The Al^3+^ signal at a binding energy of BE = 75.8 eV initially dominates and gradually decreases in intensity with increasing sputter cycles concomitantly with the O 1s signal at BE = 532 eV, while the Al^0^ signal intensity at BE = 72.8 eV increases with sputtering time due to the decreasing attenuation effects from the remaining oxide layers. The Al^3+^, Al^0^, and O 1s binding energies remain constant during the sputter cycles for the blank, 2 and 54 h samples, while for the 168 h sample the Al^3+^ and O signals were initially at higher BE and gradually shifted to lower values as the oxide layer got thinner. At the same time, the 168 h sample shows the strongest incorporation of Cl and N into the oxide layer (Figures 2c and S3c). There is a concentration gradient visible for Cl that extends from the outer oxide layers, which have been in direct contact with the electrolyte, to about 4 nm into the oxide. A similar gradient is seen for N. Here, it is interesting to note that while the BE of the N species in the weakly bound outer layers (red spectrum, BE = 402 eV) is characteristic for ammonium, the shift to lower BE in the oxide (BE = 399 eV) points to incorporation as nitride. Coming back to the shifts of the Al^3+^ and O signals for the 168 h sample, we may conclude that this results from additional bonding of Cl and N to the Al centers (e.g., formation of oxychlorides and oxynitrides). However, similar shifts might occur due to charging effects in the thicker insulating oxide layer, which cannot be completely excluded. Incorporation of Cl is also observed for the 2 and 54 h samples, although to a much lesser extent. Finally, we note that only after 168 h of soaking, Cu and Zn were clearly detectable with XPS. Corresponding SEM images of the sputtered areas show that while Al was sputtered away completely, particles consisting of Zn, Cu, and other metals were still present at the surface (Figure S4).

**Figure 3:**
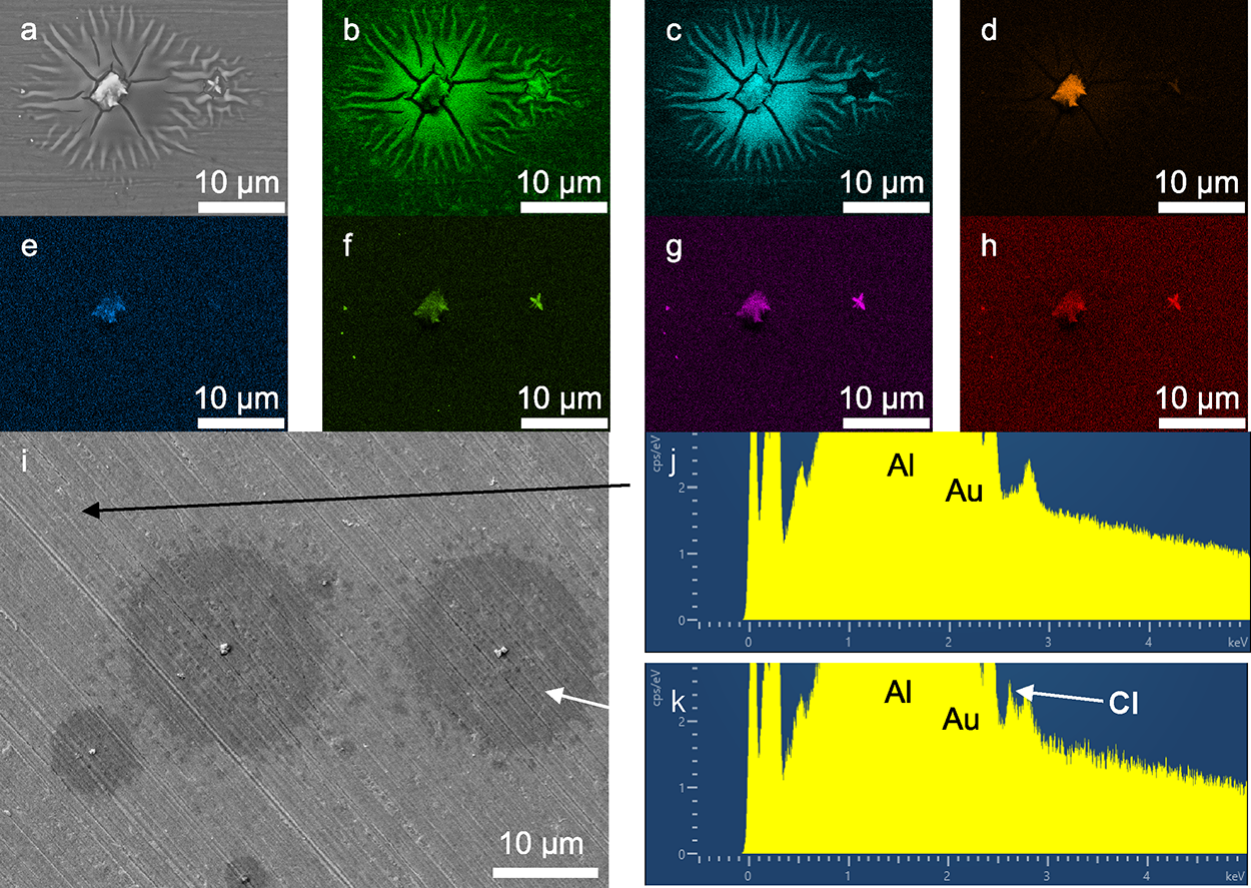
(a) SEM secondary electron image of the sample not protected from moisture after 54 h of immersion in Uralumina 150 with two large (image center) and three small (left image side) metal deposits surrounded by wrinkled Al oxide and corresponding EDS maps (acceleration voltage 10 kV) for (b) O, (c) Cl, (d) Zn, (e) Pb, (f) Cu, (g) Ga, and (h) Ni. (i) Secondary electron image of Al surface after 54 h of immersion that had been coated with a thin Au layer after immersion to protect it from moisture during transfer (acceleration voltage 10 kV). ED spectrum (j) of light area (black arrow in Figure 3i) and (k) of the dark area surrounding the particles (white arrow in Figure 3i).

The sampling area of XPS has a diameter of several millimeters and consequently, the results are averaged over a large area. In order to obtain local and highly resolved elemental compositions of the oxide layer, samples were studied by electron microscopy. Although Al was vapor-deposited on a smooth silicon wafer, a surface roughness in the nanometer range could still be observed in the TEM. The projective character of TEM imaging causes a broadening of the otherwise step-wise transitions between the sample regions. The measurements were performed on sufficiently flat sample regions. Figure 2b shows maps of Cl, Al, and O acquired by energy dispersive X-ray spectroscopy (EDS). The dashed yellow and red lines indicate the boundaries of the oxide layer identified by the electron energy loss near edge structure (ELNES) of Al^3+^. Since Au is only used as a protective layer, the corresponding EDS map is not shown. It has to be noted that Cl is quantified from the Kα peak (2.621 keV), which lies in between the Au peaks Mγ (2.409 keV) and M2N4 (2.797 keV) as shown in the SEM EDX spectra in Figure 3k. The presence of Au causes a high background, which can produce erroneous signals of other elements. Since high purity Au was used for vapor deposition, the Cl signal measured inside the Au layer was neglected. Near the oxide surface, the chlorine and aluminum contents are increased (Figure 2b). This indicates the presence of chloroaluminates inside the oxide layer. In contrast to the Cl gradient, which was present at every sample position, Zn was found on the oxide surface occasionally in some measurements after long periods of soaking. The Zn concentration in the high-purity electrolyte has been determined by inductively coupled plasma mass spectrometry (ICP-MS) to be 19 ± 4 ppm (vide infra). Obviously, Zn accumulates at the oxide–electrolyte interface. Zn was frequently found also in deposits of trace metal impurities (see Section 3.3). Figure S5 shows EDS line scans across the oxide layer including the elements Al (K-series), O (K-series), and Cl (K-series) in atom percent (at %). The thickness of the oxide layer increases by some 10 to 30% upon soaking in the chloroaluminate electrolyte. However, it has to be kept in mind that TEM measurements are highly localized and cannot represent the entire sample. No chlorine is present in the sample that was not immersed in the electrolyte. As the immersion time increases from 2 and 54 h to 168 h, the chlorine content increases. Within the oxide layer of every sample, the chlorine concentration is highest at the surface and drops toward the oxide–aluminum interface.

### 3.3 Pitting

Deposits of Ga, Cu, Pb, Ni, Ag, Fe, and Zn were found on the surface of soaked samples (Figure 3a–h). All of these elements have electrochemical standard potentials more positive than that of Al (−1.66 V). The aluminum surface thus acts as a sink for such trace metal ion impurities in the electrolyte. One of these particles and the surrounding surface was cut with a FIB and the cross-section characterized by TEM-EDS (Figure S6) in order to analyze the internal element distribution. The point of nucleation is probably a disruption of the oxide, visible in the EDS maps (orange arrows in Figure S6c,d,f,g). The bulk of the particle consists of O, Zn, and Cl with some inclusions of Ag. Some of the deposited particles have a dendritic morphology (Figure S7) and on samples that had not been protected with an Au layer, they are surrounded by a “wrinkled” surface (Figure 3a). EDS confirms that this wrinkled morphology consists of aluminum, oxygen, and a significant amount of chlorine (Figure 3b,c). The secondary electron (SE) image (Figure 3a), as well as the Cl EDS map (Figure 3c), shows that the wrinkled layer is present only on top of the big particle in the center and not on the smaller one on the right-hand side. The map also indicates that the Cl content is highest within this modified wrinkled layer. FIB-TEM results show that the alumina layer around such deposits is swollen up to a thickness of 100 nm from initially 4 nm, if no gold protection layer is applied (Figure S8a). It was also possible to establish a correlation with the immersion period. In the course of 2–18 h, both the number of chemically deposited metal particles and the fraction of wrinkled surface area increases (Figure S8b–d). Aluminum samples that had been coated with a protective Au layer to minimize the influence of humidity, during the transfer of the sample from the glove box to the microscope, did not show any wrinkling but instead a darkened area around the trace metal deposits (Figures 3i and S6a). This contrast change is attributed to a higher Cl content, which was measurable by EDS (Figure 3j,k). Wrinkling and expansion of the aluminum surface are therefore likely to be caused by the hydrolysis of chloroaluminates that have permeated the oxide layer around the trace metal deposits. The absence of this phenomenon on Au-coated samples demonstrates the efficiency of this protective measure against artifacts caused by ambient conditions. Unfortunately, galvanostatic discharge/charge cycling of aluminum electrodes does not produce a uniform deposit of aluminum on the anode even after extended periods of soaking but results in large isolated crystallites distributed statistically across the surface (Figure S9). Thus, pitting of the aluminum anode causes a highly nonuniform current distribution and thereby strongly compromises the cyclability of the battery.

### 3.4 Model of the Soaking Process and Electrochemical Impedance Monitoring

**Figure 4:**
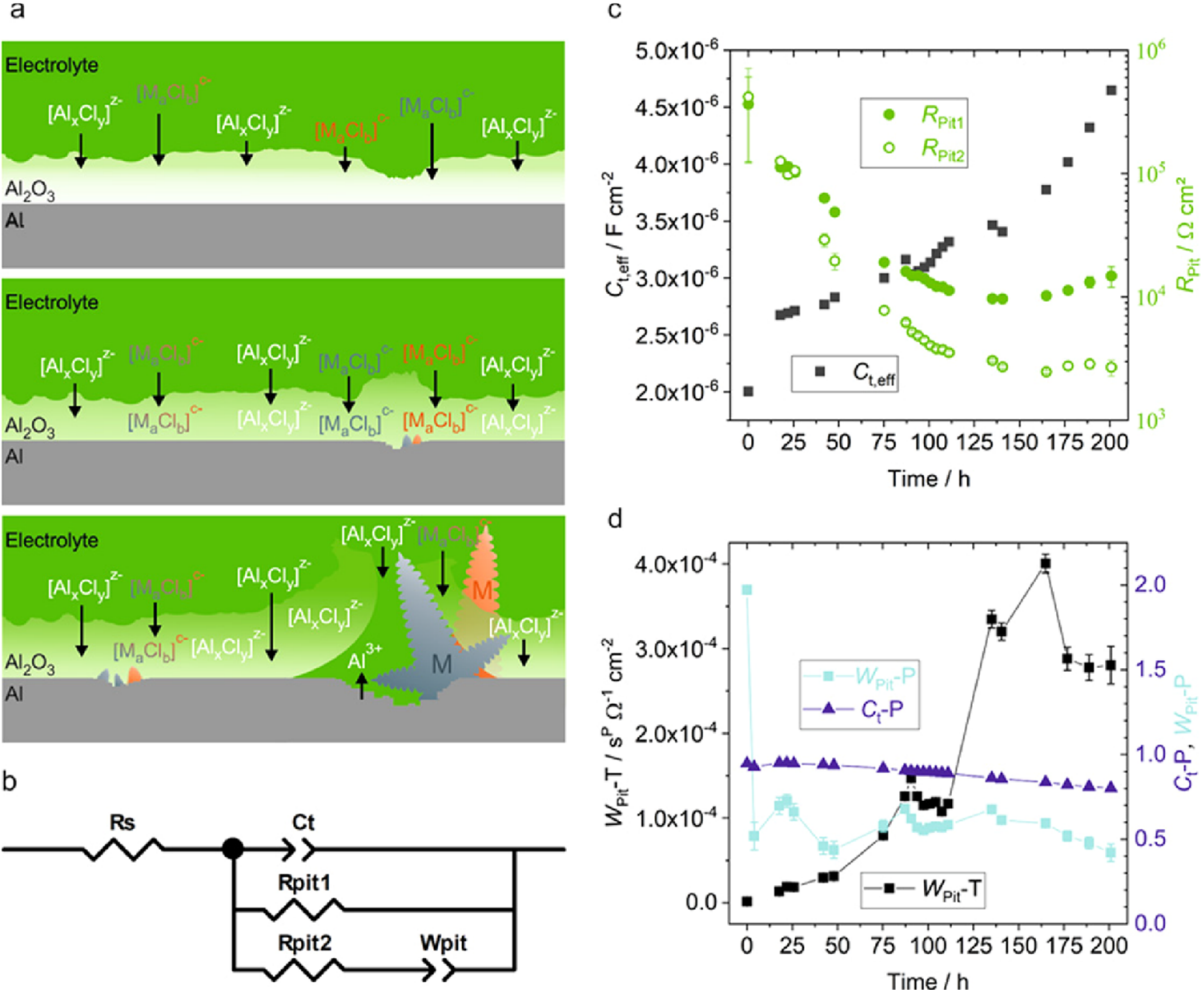
(a) Scheme of the processes (see the text) occurring during soaking of native oxide-covered Al in Uralumina 150 at ocp with increasing immersion time from top to bottom. (b) Equivalent circuit of pitting aluminum surfaces used for fitting of electrochemical impedance spectra based on Mansfeld et al.^40,43^ Progressions during soaking of (c) the total capacitance of the surface film, *C*_t,eff_, the polarization resistance of the nonpitted surface film, *R*_pit1_ and of the pitted area, *R*_pit2_, and (d) transmission line impedance *W*_pit_ in terms of the magnitude *T* and exponent *P* of a constant phase element together with the exponent *C*_t_-*P* of the constant phase element *C*_t_. Lines between the data points are meant to serve as a guide for the eye.

The combined results of ocp measurements, SNMS, XPS, and TEM/EDS provide the basis for a physico-chemical model that describes the processes occurring during soaking of aluminum electrodes in chloroaluminate electrolytes. Despite the high purity of the AlCl_3_ (99.99%, Figures S10–S14) and the urea (99.5%, Figure S15) used to produce the Uralumina 150, there are a range of metal ion impurities in the electrolyte present at ppm level. The certificates of analysis state only a small and variable number of metal impurities at low ppm levels that vary significantly from batch to batch. We thus have analyzed the electrolyte by ICP-MS for some more elements and found 19 ± 4 ppm of Zn, 1.0 ± 0.7 ppm of Fe, 1.2 ± 0.1 ppm of Ni, and 0.036 ± 0.012 ppm of Pb among others (Table S1). The speciation of these impurities is not known, but presumably such metal ions also form chlorometalates. Consequently, chloroaluminate together with impurities of other chlorometalates initially permeate the native oxide layer, and the passivity of the aluminum surface decreases (Figure 4a). Once the chlorometalate impurities reach the oxide–aluminum interface, they are chemically reduced by the less noble aluminum and the foreign metals nucleate. The oxidized aluminum ions react with the released chloride ions and surrounding chloroaluminates. A corresponding redox reaction is shown here for the example of zinc in eq 5.
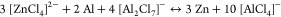
5

Growth of the nuclei and concomitant underetching of the oxide layer probably lead to blistering and cracking and consequently to pitting corrosion at a few sites statistically distributed over the electrode surface. A large fraction of the aluminum surface, however, remains covered by the permeated oxide film. The ocp traces shown in Figure 1a thus indicate a changing mixed potential of the cathodic deposition of trace metals like Zn, Fe, etc., becoming rate limiting over time because of depletion and the anodic dissolution of Al from an increasingly active surface. These processes occur to a variable extent (due to the abrasion pre-treatment) also on the Al wire reference electrode, which explains why it occasionally had a potential slightly more positive than the Al working electrode.

The soaking period has also been monitored by EIS. A typical progression of such spectra of an Al electrode in a three-electrode glass cell recorded at certain soaking intervals at ocp is shown in Figure S16. The impedance changes over many orders of magnitude between the very highest frequencies to that at the very lowest frequencies, and thus, the EIS data are best displayed as Bode plots in which the magnitude of the logarithm of the impedance modulus and the phase angle are plotted vs the logarithm of the applied frequency.^39^ The spectra in a frequency range of 100 kHz to 1 mHz show two-time constants and resemble the impedance behavior found for oxide-covered aluminum in aqueous chloride solution.^7^ The different stages of the corrosion process shown in Figure 4a do not progress sequentially in a uniform manner across the Al surface. Immediately after immersion into the electrolyte, chloroaluminate species and chlorometalate impurities start to permeate the oxide film. As a result, a composition gradient develops in the layer as indicated by the EDS line scans in Figure S5, and the dielectric properties of the oxide film change gradually. Initially, this gradient is strong, and thus, the diffusional rate is high. Consequently, the electrochemical impedance of the layer changes over immersion time with decreasing rates. Since the thickness of the native oxide film varies locally between 3 and 4 nm (observed by TEM), also the period varies in which the permeating electrolyte species reach the oxide–aluminum interface and the more noble metals progressively nucleate statistically distributed at the oxide–aluminum interface. The resulting aluminum surface with electrochemically active pits resembles a partially blocked electrode, which can also be described as an array of microelectrodes. Again, this stage is not stationary but changes over immersion time as an increasing number of pits are forming. The impedance behavior of a pitting aluminum surface in aqueous chloride solution has been modeled with an equivalent circuit described by Mansfeld et al.^40^ (Figure 4b). *R*_S_ stands for the electrolyte resistance and *C*_t_ (implemented as a constant phase element) for the total capacitance of the surface film. The latter cannot be divided into the capacitance of the pits (area *F*) and that of the nonpitted area (1 – *F*) because they are exactly in parallel and thus indistinguishable. *R*_pit1_ represents the quantity *R*_p_/(1 – *F*), where *R*_p_ is the polarization resistance of the nonpitted surface film. *R*_p_ can only be calculated directly, if the fraction of the surface covered by pits (*F*) is very small and consequently, *R*_pit1_ ≅ *R*_p_/(1 – *F*). *R*_pit2_ represents the polarization resistance of the pitted area. *W*_pit_ is a transmission line impedance, which models the mass transport at the active pits in terms of a constant phase element. This model produces acceptable fits of the data from nonaqueous chloroaluminate electrolytes only up to a certain period of soaking (Figure S17). After about 1 week of immersion, the quality of the fit becomes worse (Figures S18 and S19), which is thought to be the result of processes not accounted for in the equivalent circuit, e.g., significant underetching and partial detachment of the permeated layer around the pits. Likewise, the very first spectrum recorded immediately after immersion into the electrolyte cannot be fitted over the whole frequency range, but only down to about 0.3 Hz (Figure S20). The initial changes of the surface layer are too fast to treat the sample as a quasi-stationary system. This is obvious also from the ocp traces shown in Figure 1a, where the potential decreases rapidly over the first 3 to 4 h, corresponding to the measurement period of one impedance spectrum. The results of the analysis are depicted in the two diagrams in Figure 4c,d. The solution resistance *R*_S_ corresponding to the *x* axis intercept at the high frequency end of the spectra in the Nyquist plots is 82 ± 12 Ω·cm^2^ and not shown in these graphs. The resistances *R*_pit1_ and *R*_pit2_ are initially large and quite similar. During soaking, the resistances of the nonpitted and the pitted active areas decrease, with the latter assuming values one order of magnitude smaller than that of the former. In other words, the pitted area increases and becomes more active with soaking. This is also reflected in the increase of the transmission line impedance *W*_pit_-*T*, where the exponent *W*_pit_-*P* of the constant phase element varies around 0.5, indicating a Warburg impedance caused by diffusive transport of electroactive chloroaluminate species at the pits. The total effective capacitance *C*_t,eff_ of the layer has been calculated from the parameter *C*_t_-*T* and the exponent *C*_t_-*P* of the constant phase element *C*_t_ with eq 6,
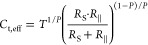
6where *R*_∥_ = 1/(1/*R*_Pit1_ + 1/*R*_Pit2_).^41,42^ The exponent *P* shown in Figure 4d is initially around 0.95 and after 2 days of soaking starts to slowly drop toward 0.80. In the beginning of soaking, the CPE thus behaves almost like an ideal capacitance, but permeation of electrolyte changes this behavior. The dielectric constant ε of the oxide film can be calculated from *C*_t,eff_ with eq 7 assuming an average thickness δ of 4 nm, which has been measured by XPS and STEM.
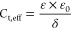
7

This produces a dielectric constant of 9 for the initial capacitance of the nonpermeated film and agrees well with values found in the literature for Al_2_O_3_.^10^ During soaking, *C*_t,eff_ increases continuously and after 1 week reached a value of 3.8 × 10^–6^ F·cm^–2^. Assuming a film thickness of 6.7 nm (measured by XPS after 1 week of soaking) and neglecting the increased pitted area, the dielectric constant of the film has increased to a maximum value of 29. Although the dielectric constant of the chloroaluminate electrolyte is not known, it probably is significantly higher than that of Al_2_O_3_, and the observed increase of the total effective capacitance is thus caused by the uptake of chloroaluminates in the film in addition to the increasing active area caused by pitting. Altogether, the progression of the electrochemical impedance spectra recorded during soaking of the native oxide-covered aluminum agrees well with the results of ocp measurements and ex situ characterization of the oxide layers by SNMS, EDS/TEM, as well as EDS/SEM.

## 4 Conclusions

Aluminum has been suggested as an anode material for rechargeable batteries with chloroaluminate electrolytes. Aluminum–sulfur cells are usually assembled in the charged state; i.e., initially, the battery is discharged with dissolution of aluminum at the anode. The native oxide on the aluminum anode causes high overpotentials during the initial cycles and thus compromises the voltage efficiency of the battery. Pre-immersion of the passive Al anode in chloroaluminate electrolytes has been reported to diminish the passivation. The processes occurring upon immersion of native oxide-covered aluminum in chloroaluminate DESs have been studied in order to understand the fate of the passive layer. The decrease of the ocp of the passive aluminum during soaking in the electrolyte indicates an activation of the Al surface. SNMS shows uptake of chloroaluminates into the oxide layer, which scales with the length of the soaking period. Analyses of XPS depth profiles correlate the penetration depth of chlorine-containing species to the soaking period and show the built-up of a concentration gradient of chloroaluminates in the oxide film. The average thickness of the oxide layer increases from 3.8 to 6.7 nm during soaking. Permeation of nitrogen-containing species, i.e., urea, was seen after extended periods of immersion. Chemical reactions between the oxide and the electrolyte species could not be confirmed. Formation of a concentration gradient of chloroaluminate species in the oxide layer upon immersion in the electrolyte is also detected locally with EDS in the TEM. Extensive chloroaluminate permeation of the native oxide layer is considered highly beneficial for the discharge/charge behavior of the battery because it can result in a uniform activation of the aluminum surface. However, SEM images after soaking show local chemical deposits of foreign metals at pits originating from chlorometalate impurities present in the electrolyte. The electrochemical impedance spectra measured during a soaking period of 8 days could be fitted to an equivalent circuit model originally developed for pitting of passive aluminum in aqueous chloride solution. The increasing total effective capacitance and decreasing interfacial resistance reveal a continuous activation of the anode. SEM images after discharge–charge cycles of aluminum electrodes in symmetrical battery cells with chloroaluminate electrolytes show local dissolution and deposition of aluminum preferentially at the active pits. This nonuniform current density distribution as a result of pitting is highly detrimental to the cyclability of secondary aluminum batteries. In order to mitigate these effects, the passivity of aluminum anodes should be minimized before cell assembly and the purity level of the electrolyte increased.

## Supporting Information Available

SEM images of Al electrodes after Al deposition from Acetlumina 150 and from a 1.5:1 mixture of AlCl_3_ and 1-ethyl-3-methylimidazolium chloride; elemental depth profiles of the samples after different immersion periods measured by SNMS; XPS sputter profiles of Al, O, and N of samples soaked for 0, 2, 54, and 168 h in Uralumina 150; SEM secondary electron images of a sample immersed for 168 h after complete removal of Al layer by sputtering in the XPS; EDS line scans showing the Al, O and Cl content in different regions of the Al oxide layer after different immersion periods; SEM image of a gold-covered impurity deposit on an Al thin film electrode after 168 h of immersion analyzed by TEM; HAADF SE image of the impurity deposit and corresponding TEM-EDS maps of Zn, Al, Ag, Cl, O and Au; SEM image of dendritic metallic impurity chemically deposited on hp Al surface after soaking in Uralumina 150 at ocp; EFTEM image of the oxygen edge on high purity Al after immersion in electrolyte and sample transfer under ambient conditions and SEM images of the surface of high purity Al after immersion periods of 2, 6, and 18 h; SEM image of aluminum anode after 54 h of soaking followed by two galvanostatic discharge/charge cycles in a symmetrical Swagelok type battery cell with Uralumina 150 electrolyte; certificates of analysis by the supplier of AlCl_3_ and urea; ICP-MS analysis of a blank sample of ultrapure water and a batch of Uralumina 150 electrolyte; electrochemical impedance spectra of a native oxide-covered aluminum electrode in a glass cell during various periods of soaking (0–201 h) in Uralumina 150; electrochemical impedance spectra of a native oxide-covered aluminum electrode recorded at open circuit potential after 0, 18, 135, and 168 h of soaking in Uralumina 150 (PDF)
