# Supplementary material for: Corrosion of Passive Aluminum Anodes in a Chloroaluminate Deep Eutectic Solvent for Secondary Batteries: The Bad, the Good, and the Ugly
Source: ACS Appl Mater Interfaces. 2022 Dec 27;15(1):882–92. doi: 10.1021/acsami.2c16153 (PMC9837816; doi:10.1021/acsami.2c16153)
Supplement: Supplementary file 1 — am2c16153_si_001.pdf [file am2c16153_si_001.pdf]

Supporting Information:

Corrosion of Passive Aluminum Anodes in a Chloroaluminate Deep Eutectic Solvent for Secondary Batteries — the Bad, the Good, and the Ugly

David Moser[a], Philipp Materna[b], Anna Stark[b], Judith Lammer[c], Attila Csík[d], Jasmin M. Abdou[e], Raphael Dorner[e], Martin Sterrer[e], Walter Goessler[f], Gerald Kothleitner[a,c], Bernhard Gollas[b,]*

[a]Institute of Electron Microscopy and Nanoanalysis, Graz University of Technology, Steyrergasse 17, 8010 Graz, Austria

[b]Institute for Chemistry and Technology of Materials, Graz University of Technology, Stremayrgasse 9/II, 8010 Graz, Austria

[c]Graz Centre for Electron Microscopy, Steyrergasse 17, 8010 Graz, Austria

[d]Institute for Nuclear Research, Bem ter 18/c, 4026 Debrecen, Hungary

[e]Institute of Physics, University of Graz, Universitätsplatz 5, 8010 Graz, Austria

[f]Institute of Chemistry, University of Graz, Universitätsplatz 1, 8010 Graz, Austria

bernhard.gollas@tugraz.at

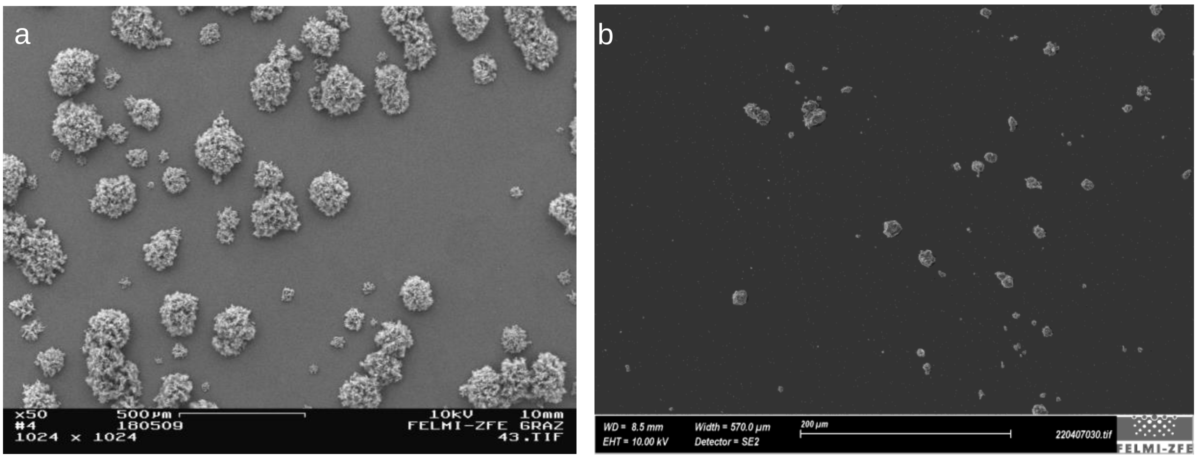

Figure S1. SEM images of Al electrodes **(a)** after 1 h of galvanostatic Al deposition (0.217 mA·cm^{-2}) in a symmetrical Swagelok type battery cell with the DES Acetlumina 150 and **(b)** after 0.5 h of galvanostatic Al deposition (0.217 mA·cm^{-2}) with a 1.5:1 mixture of $AlCl_3$ and 1-ethyl-3-methylimidazolium chloride.

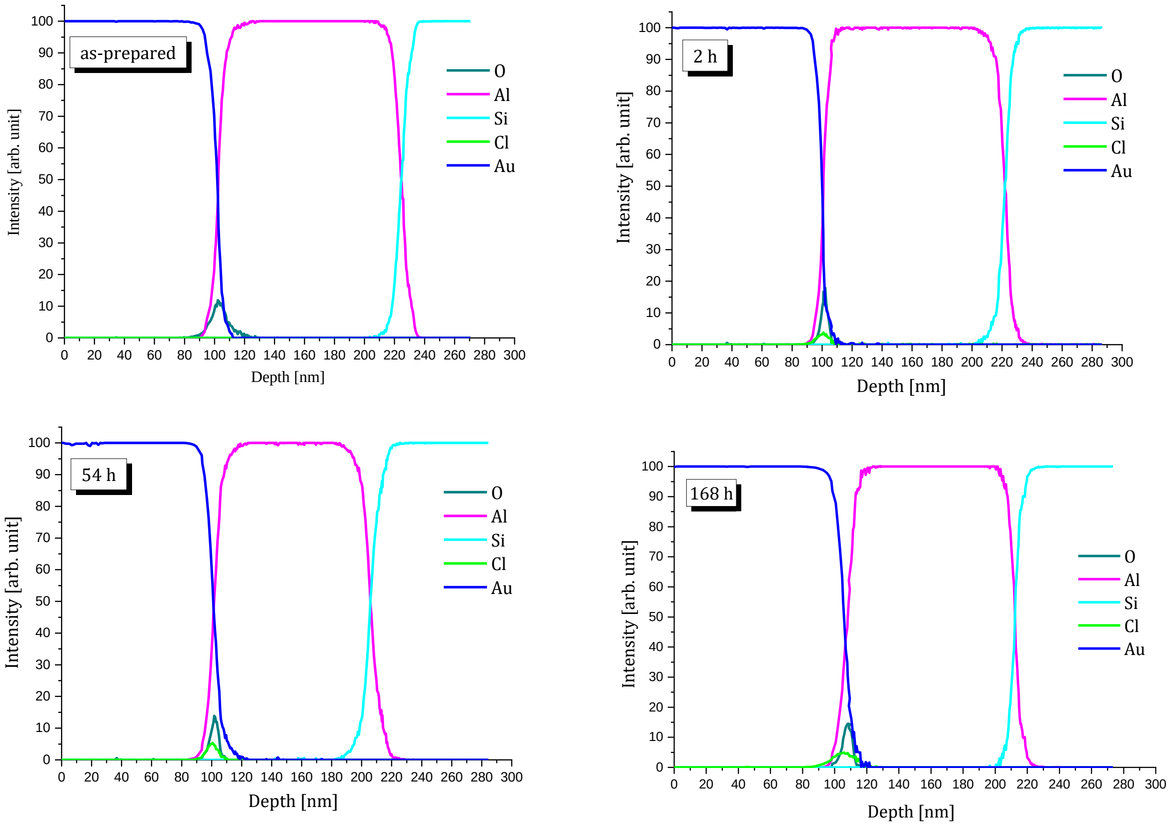

Figure S2. Elemental depth profiles of the samples after different immersion periods (see legends) measured by Secondary Neutral Mass Spectrometry (SNMS).

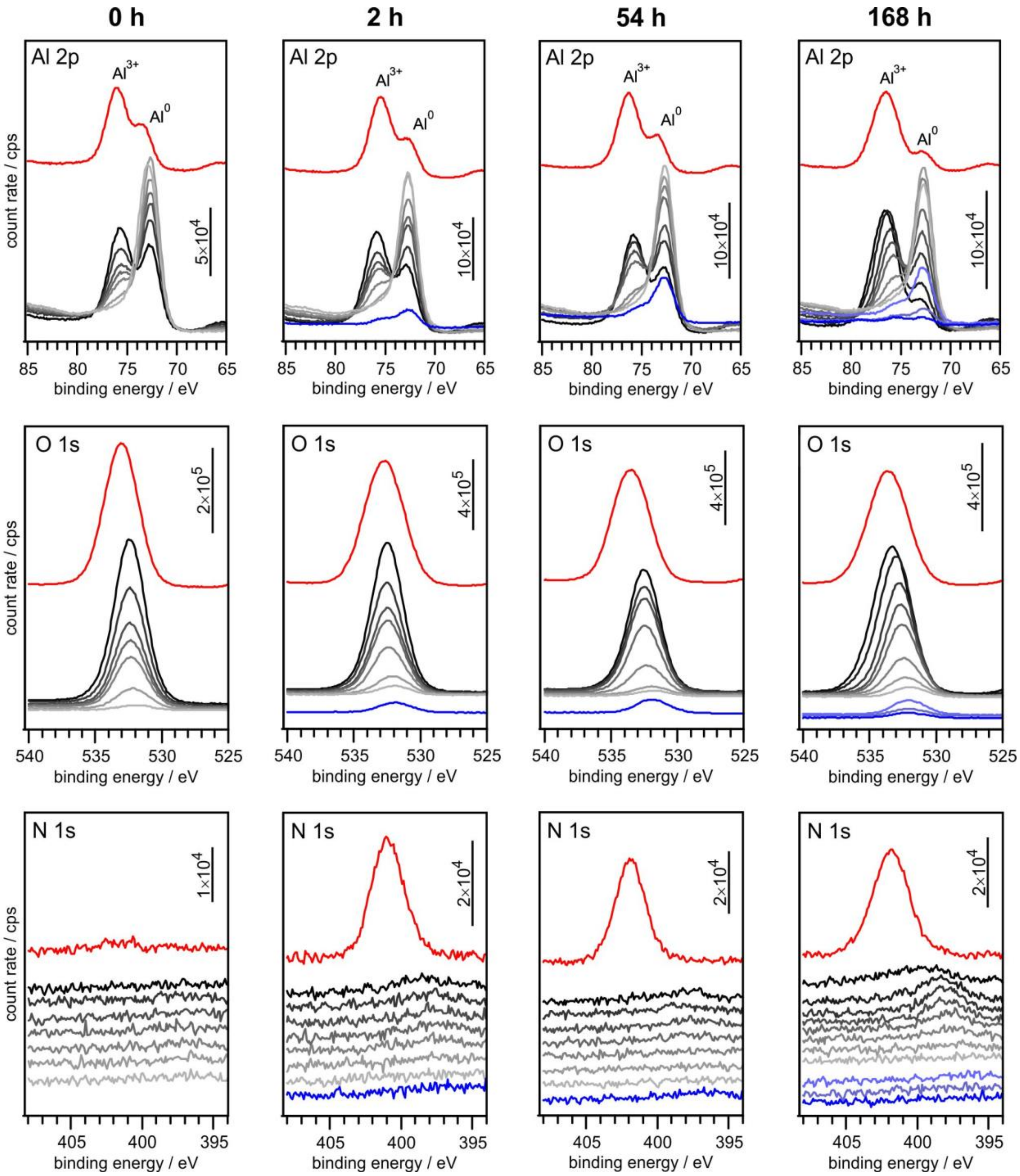

Figure S3. XPS sputter profiles of Al (top row), O (middle row) and N (bottom row) of pristine samples (0 h of soaking) and samples soaked for 2 h, 54 h, and 168 h in Uralumina 150 (red: as-received sample; black-gray: progressive change in Al film with increasing number of sputter cycles. blue: after almost complete removal of Al film).

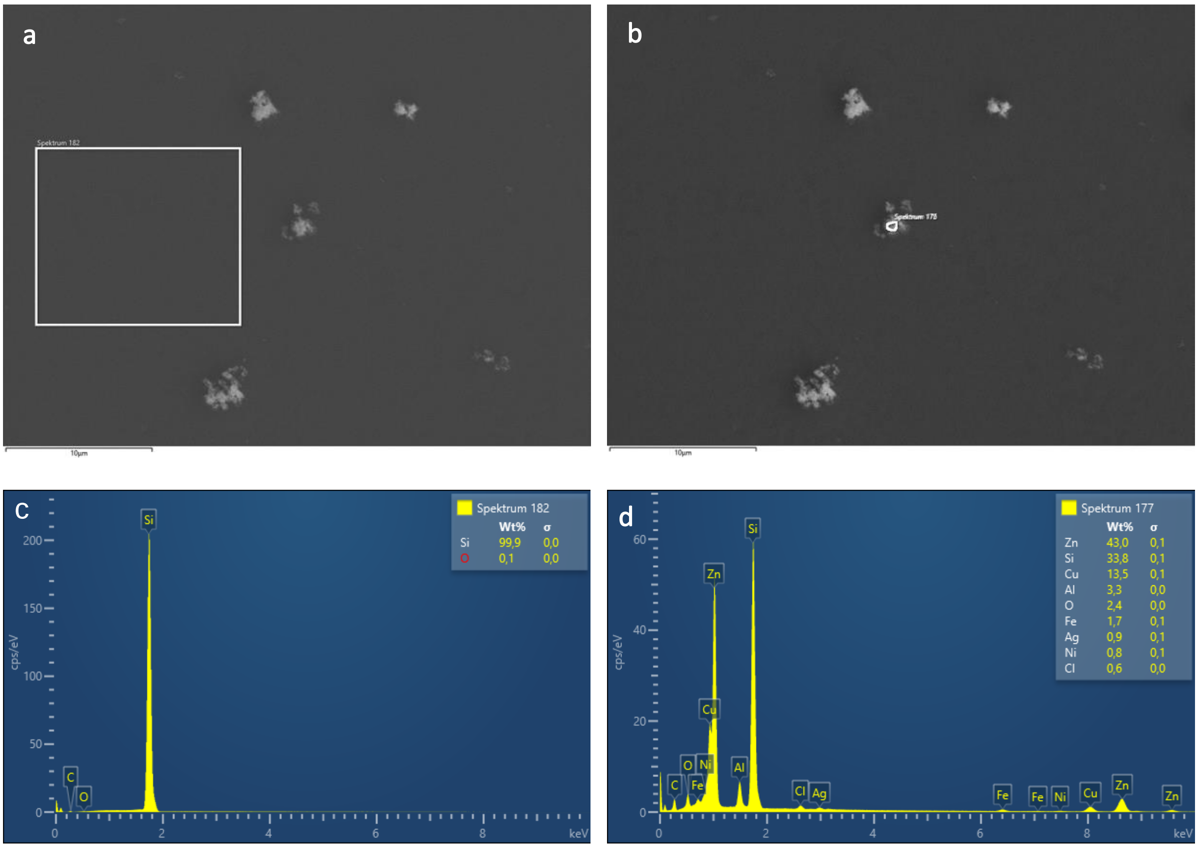

Figure S4. SEM secondary electron images (acceleration voltage 14 kV) of a 168 h immersed sample after complete removal of Al layer by sputtering in the XPS. White frames indicate areas probed by EDS of **(a)** silicon substrate laid bare and **(b)** incompletely sputtered impurity deposit. EDS spectrum of **(c)** framed area in **(a)** and **(d)** framed particle in **(b)**.

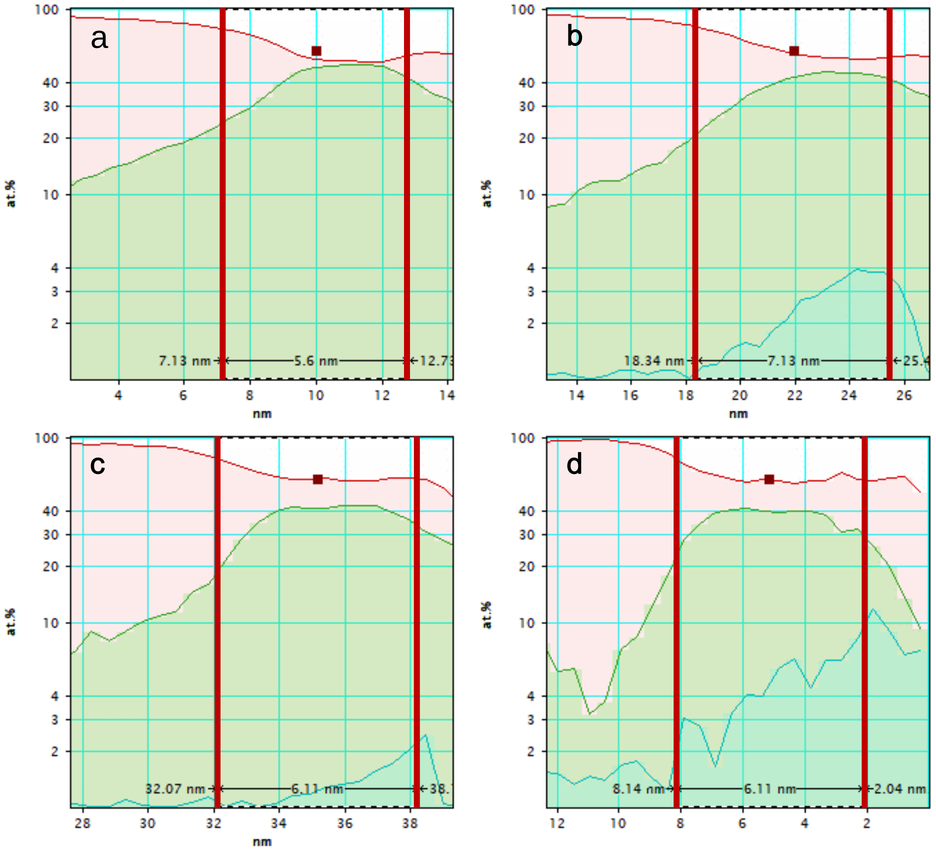

Figure S5. EDS line scans showing the Al (red; 40-100 %; K-series), O (green; 10-40 %; K-series) and Cl (cyan; 0-10 %; K-series) content in different regions of the Al oxide layer after different immersion periods: **(a)** 0 h, **(b)** 2 h, **(c)** 54 h and **(d)** 168 h. Red lines indicate the border of the oxide layer, which were determined using the ELNES of Al^{3+}.

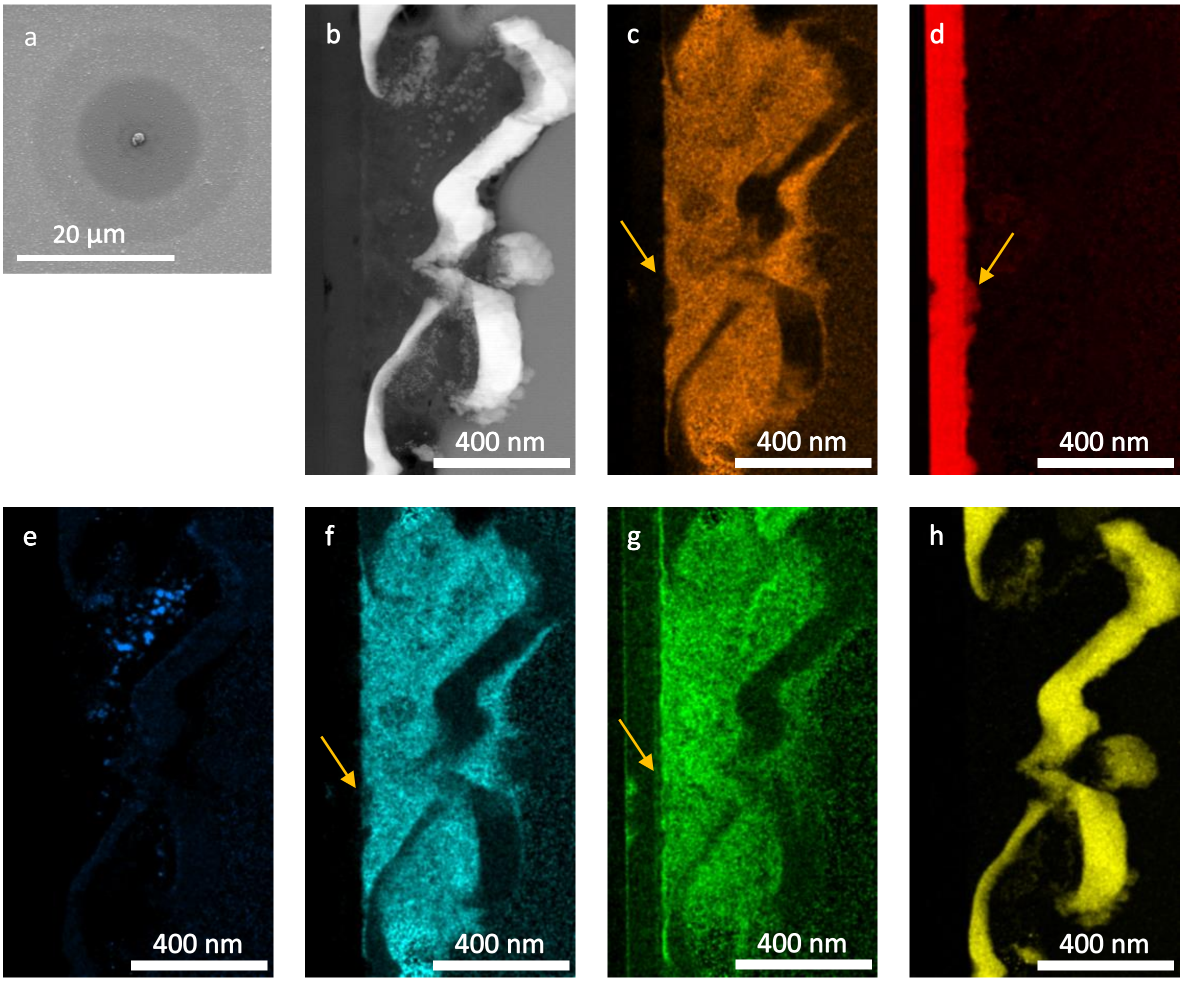

Figure S6. (a) SEM image of a gold covered impurity deposit on an Al thin film electrode after 168 h of immersion analysed by TEM. **(b)** HAADF SE image of the impurity deposit and corresponding TEM-EDS maps of **(c)** Zn, **(d)** Al, **(e)** Ag, **(f)** Cl, **(g)** O, **(h)** Au.

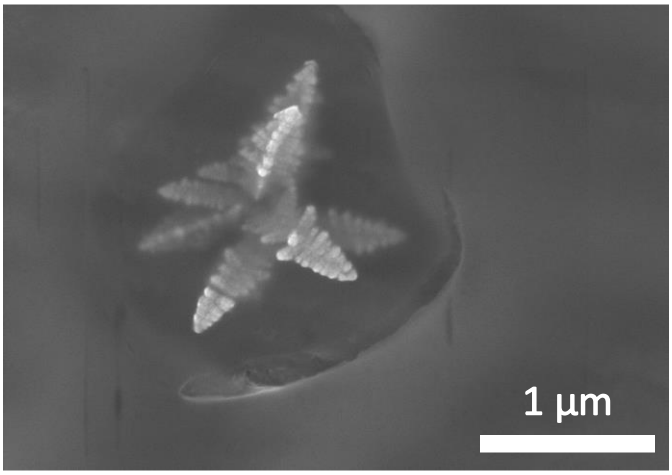

Figure S7. SEM image of dendritic metallic impurity chemically deposited on hp Al surface after soaking in Uralumina 150 at ocp.

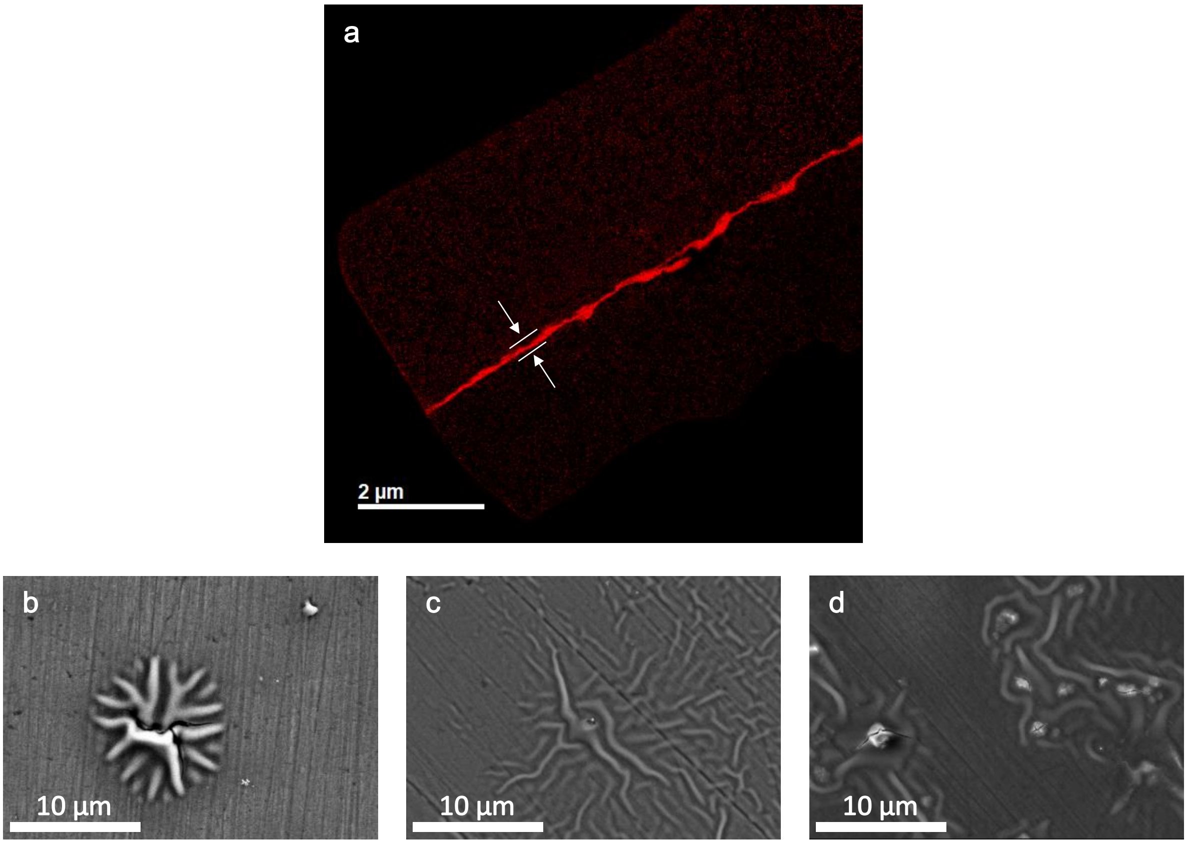

Figure S8. (a) Energy Filtered Transmission Electron Microscope (EFTEM) image of the oxygen edge on high purity Al after immersion in electrolyte and sample transfer under ambient conditions and SEM images of the surface of high purity Al after immersion periods of **(b)** 2 h, **(c)** 6 h, and **(d)** 18 h.

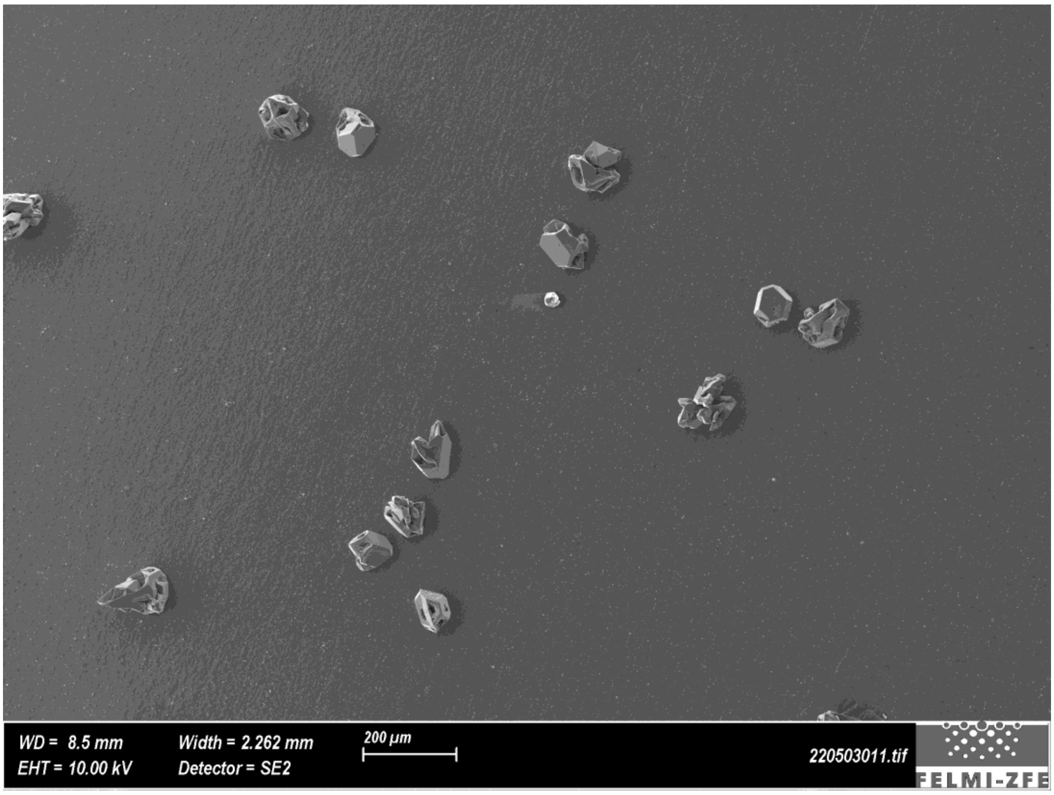

Figure S9. SEM image of aluminum anode after 54 h of soaking followed by two galvanostatic discharge/charge cycles (0.217 mA·cm^{-2}, 1 mA·h·cm^{-2}) in a symmetrical Swagelok type battery cell with Uralumina 150 electrolyte.

Certificate of Analysis

**Product name:**	**Aluminum chloride, anhydrous; 99.99%-Al**
**Product no.:**	**AB119614**
**CAS no.:**	**[7446-70-0]**
**Lot no.:**	**1375329**

**Test**	**Result**
Appearance	white to yellow powder
Metals purity	99.99%

**Element**	**ppm**
Fe	7

Oksana Grübel
Product Safety Manager

Phone +49 721-95061-591
Fax +49 721-95061-80
o.gruebel@abcr.com

Karlsruhe, 06.04.2022

This document is not intended to assure certain properties of products or their suitability for a specific purpose.
This document does not release from industry standard operational control.

[illegible] GmbH [illegible] Karlsruhe Germany — Phone +49 721 [illegible] Fax +49 721 [illegible] [illegible] — VAT no. DE [illegible] [illegible] — [illegible] Registered office Karlsruhe Managing Director [illegible]

Figure S10. Certificate of analysis of $AlCl_3$.

Certificate of Analysis

**Product name:**	**Aluminum chloride, anhydrous; 99.99%-Al**
**Product no.:**	**AB119614**
**CAS no.:**	**[7446-70-0]**
**Lot no.:**	**1409665**

**Test**	**Result**
Appearance	white to yellow powder
Assay	>99.99%

Ca	10 ppm	Mg	20 ppm
Cu	1 ppm	Si	20 ppm
Fe	2 ppm	Ti	2 ppm

M. Weber

Dipl. Chem. Martina Weber
Product Safety Manager

Phone +49 (0)721 95061 57
Fax +49 (0)721 95061 33
m.weber@abcr.de

Karlsruhe, 18.09.2020

This document is not intended to assure certain properties of products or their suitability for a specific purpose.
This document does not release from industry standard operational control.

EQ ZERT ISO 9001 CERTIFIED

abcr GmbH
Im Schlehert 10
76187 Karlsruhe
Germany

Phone +49 721 95061-0
Fax +49 721 95061-80
info@abcr.de • www.abcr.de

VAT no. DE 815518524
Tax no. 35005/14749
DUNS no. 320054844

AG Mannheim HRB 104793
Registered office Karlsruhe
Managing director Dr. Jan Schuricht

Figure S11. Certificate of analysis of $AlCl_3$.

Certificate of Analysis

Product name: **Aluminum chloride, anhydrous; 99.99%-Al**

Product no.: **AB119614**

CAS no.: **[7446-70-0]**

Lot no.: **1426376**

**Test**	**Result**
Appearance	white to yellow powder
Assay	>99.99%

Ca	1 ppm	Mg	3 ppm
Cu	<0.1 ppm	Si	1 ppm
Fe	0.4 ppm	Ti	<0.2ppm

Dr. Inna Oksengendler
Product Safety Director

Tel. +49 (0)721 95061 34
Fax +49 (0)721 95061 33
i.oksengendler@abcr.de

Karlsruhe, 21.09.2020

This document is not intended to assure certain properties of products or their suitability for a specific purpose.
This document does not release from industry standard operational control.

abcr GmbH
Im Schlehert 10
76187 Karlsruhe
Germany

Phone +49 721 95061-0
Fax +49 721 95061-80
info@abcr.de • www.abcr.de

VAT no. DE 815518524
Tax no. 35005/14749
DUNS no. 320054844

AG Mannheim HRB 104793
Registered office Karlsruhe
Managing director Dr. Jan Schuricht

Figure S 12. Certificate of analysis of $AlCl_3$.

Certificate of Analysis

**Product name:**	**Aluminum chloride, anhydrous; 99.99%-Al**
**Product no.:**	**AB119614**
**CAS no.:**	**[7446-70-0]**
**Lot no.:**	**1447365**

**Test**	**Result**
Appearance	white to yellow powder
Metals purity	99.99%

**Element**	**ppm**	**Element**	**ppm**
Mg	3	Ca	1
Si	1	Fe	0.4

Dipl. Ing. Ute Eschrig
Product Safety Manager

Phone +49 (0)721 95061 46
Fax +49 (0)721 95061 33
u.eschrig@abcr.de

Karlsruhe, 06.04.2022

This document is not intended to assure certain properties of products or their suitability for a specific purpose.
This document does not release from industry standard operational control.

[illegible]

Figure S13. Certificate of analysis of $AlCl_3$.

Certificate of Analysis

**Product name:**	**Aluminum chloride, anhydrous; 99.99%-Al**
**Product no.:**	**AB119614**
**CAS no.:**	**[7446-70-0]**
**Lot no.:**	**1467923**

**Test**	**Result**
Appearance	white to yellow powder
Metals purity	99.99%

**Element**	**ppm**	**Element**	**ppm**
Mg	20	Ca	5
Si	1	Fe	0.4

Oksana Grübel
Product Safety Manager

Phone +49 721-95061-591
Fax +49 721-95061-80
o.gruebel@abcr.com

Karlsruhe, 03.03.2022

This document is not intended to assure certain properties of products or their suitability for a specific purpose.
This document does not release from industry standard operational control.

abcr GmbH
Im Schlehert 10
76187 Karlsruhe
Germany

Phone +49 721 95061-0
Fax +49 721 95061-80
info@abcr.de · www.abcr.de

VAT no. DE 815518524
Tax no. 35005/14749
DUNS no. 320054844

AG Mannheim HRB 104793
Registered office Karlsruhe
Managing Director Dr. Jan Schuricht

Figure S14. Certificate of analysis of $AlCl_3$.

SIGMA-ALDRICH

3050 Spruce Street, Saint Louis, MO 63103 USA
Email USA: techserv@sial.com Outside USA: eurtechserv@sial.com

Certificate of Analysis

**Product Name:**	UREA<br>puriss. p.a., ACS reagent, reag. Ph. Eur., >= 99.5 %
**Product Number:**	33247
**Batch Number:**	STBG5842
**Brand:**	Sigma-Aldrich
**CAS Number:**	57-13-6
**Formula:**	NH_2CONH_2
**Formula Weight:**	60.06
**Quality Release Date:**	07 OCT 2016
**Recommended Retest Date:**	SEP 2020

TEST	SPECIFICATION	RESULT
ASSAY	≥ 99.5 %	100.2 %
	99-101 %(CALC. TO THE DRIED SUBSTANCE)	100.2 %
MELTING POINT	132 - 135 C	132 - 134 DEG C
INSOLUBLE MATTER	≤ 0.01 % (WATER INSOLUBLE MATTER)	COMPLYING
LOSS ON DRYING	≤ 1.0 %	0.1 %
SULFATED ASH	≤ 0.01 %	0.01 %
APPEARANCE OF SOLUTION	COMPLYING	COMPLYING
HEAVY METALS	≤ 0.0005 % (AS PB)	< 0.0005 %
PROOF OF IDENTITY	COMPLYING	COMPLYING
MISCELLANEOUS TESTS	ALKALINELY REACTING	
	UBSTANCES: COMPLYING	COMPLYING
	BIURET(CARBAMYLUREA): ≤ 0.1 %	0.1 %
AMMONIUM (NH4+)	≤ 0.05 %	< 0.01 %
CHLORIDE (CL)	≤ 0.0005 %	< 0.0005 %
SULFATE (SO4)	≤ 0.001 %	< 0.001 %
IRON	≤ 0.0002 %	< 0.0002 %

Claudia Mayer
Manager Quality Control
Steinheim, Germany

Sigma-Aldrich warrants that at the time of the quality release or subsequent retest date this product conformed to the information contained in this publication. The current specification sheet may be available at Sigma-Aldrich.com. For further inquiries, please contact Technical Service. Purchaser must determine the suitability of the product for its particular use. See reverse side of invoice or packing slip for additional terms and conditions of sale.

Sigma-Aldrich Certificate of Analysis - Product 33247 Lot STBG5842 Page 1 of 1

Figure S15. Certificate of analysis of urea.

Table S1. Concentration of selected impurities found by ICP-MS in a blank sample of ultrapure water (18.2 MΩ cm) and a batch of Uralumina 150 after 1000-fold dilution with ultrapure water.

Element	Blank sample / µg·kg^{-1}	Uralumina 150 / µg·kg^{-1}
Zn	< 1.0	19 ± 4
Fe	< 1.0	1.9 ± 0.7
Ni	< 1.0	1.2 ± 0.1
Y	< 0.01	0.37 ± 0.01
Cd	< 0.01	0.069 ± 0.004
In	< 0.01	< 0.01
Gd	< 0.01	0.049 ± 0.001
Pb	0.053 ± 0.038	0.036 ± 0.012
U	< 0.01	0.17 ± 0.01

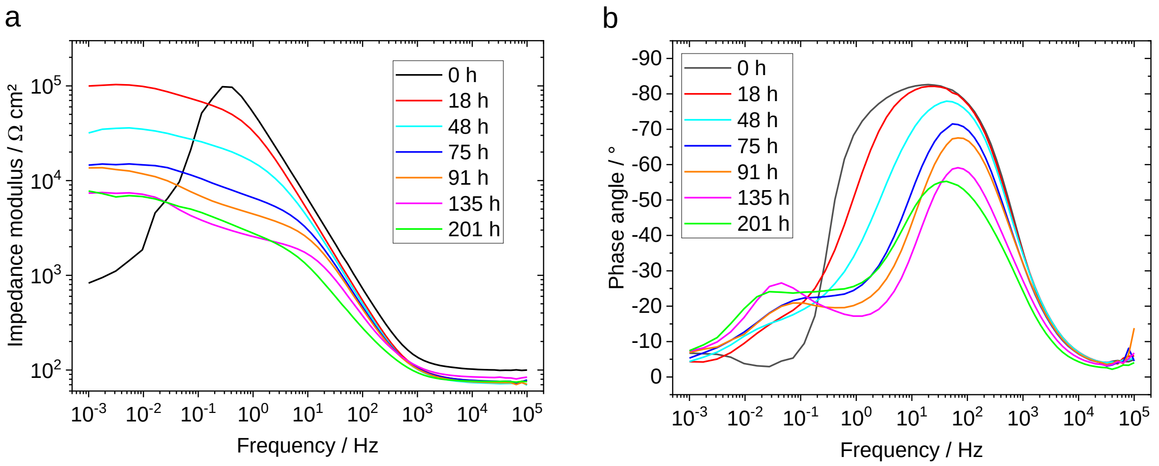

Figure S16. Typical progression of electrochemical impedance spectra of a native oxide covered aluminum electrode in a glass cell during various periods of soaking (0 to 201 h) in Uralumina 150: Bode plots with **(a)** impedance modulus and **(b)** phase angle.

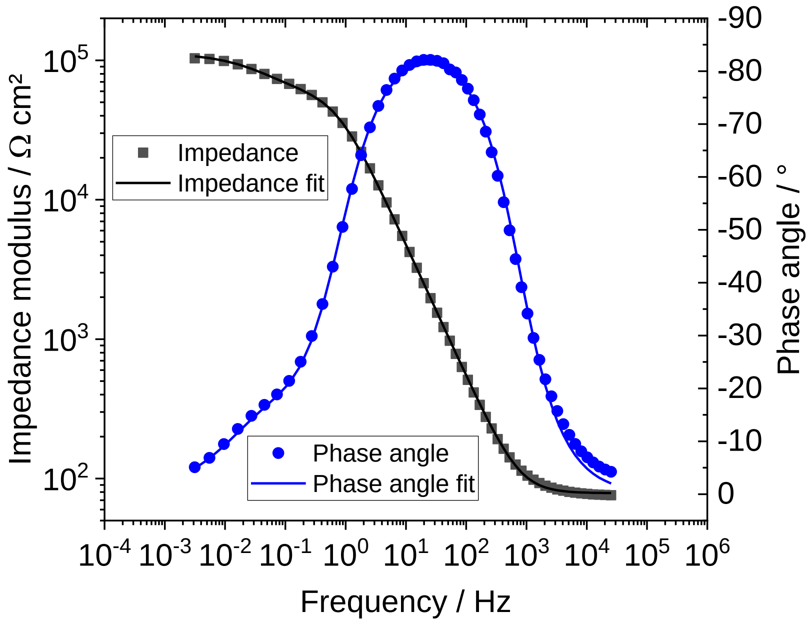

Figure S17. Electrochemical impedance spectrum (Bode plot) of a native oxide covered aluminum electrode recorded at open circuit potential after 18 h of soaking in Uralumina 150. Lines are best fits in the frequency range 25 kHz to 3 mHz to the equivalent circuit of pitting aluminum surfaces in **Figure 4b**.

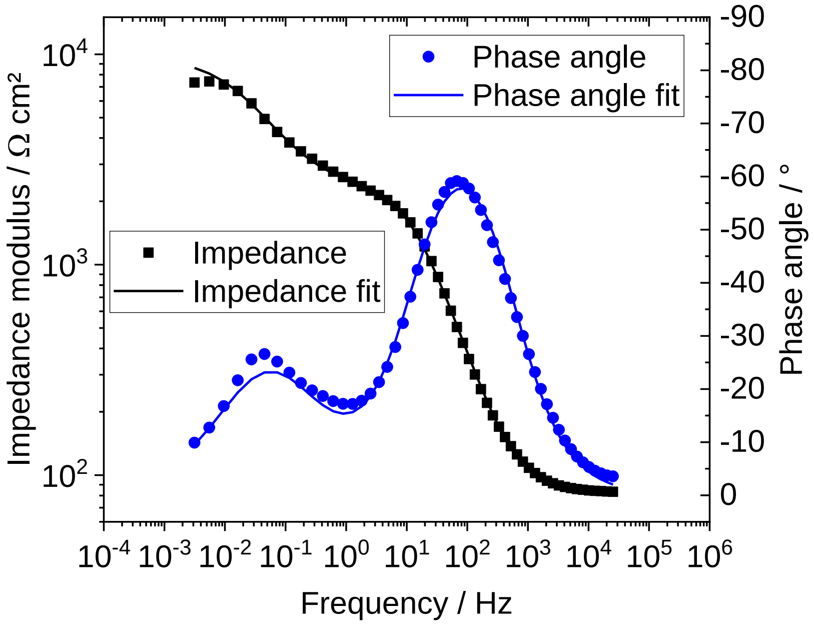

Figure S18. Electrochemical impedance spectrum (Bode plot) of a native oxide covered aluminum electrode recorded at open circuit potential after 135 h of soaking in Uralumina 150. Lines are best fits in the frequency range 25 kHz to 3 mHz to the equivalent circuit of pitting aluminum surfaces in **Figure 4b**.

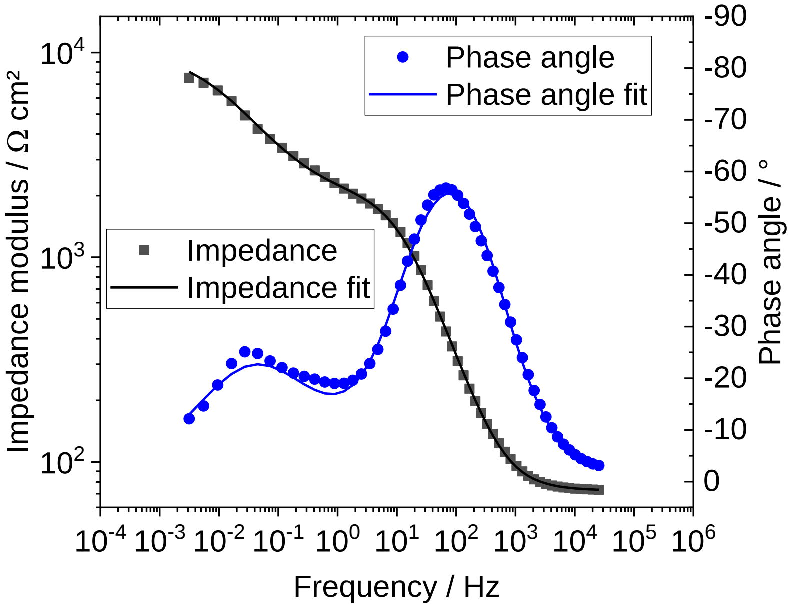

Figure S19. Electrochemical impedance spectrum (Bode plot) of a native oxide covered aluminum electrode recorded at open circuit potential after 168 h of soaking in Uralumina 150. Lines are best fits in the frequency range 25 kHz to 3 mHz to the equivalent circuit of pitting aluminum surfaces in **Figure 4b**.

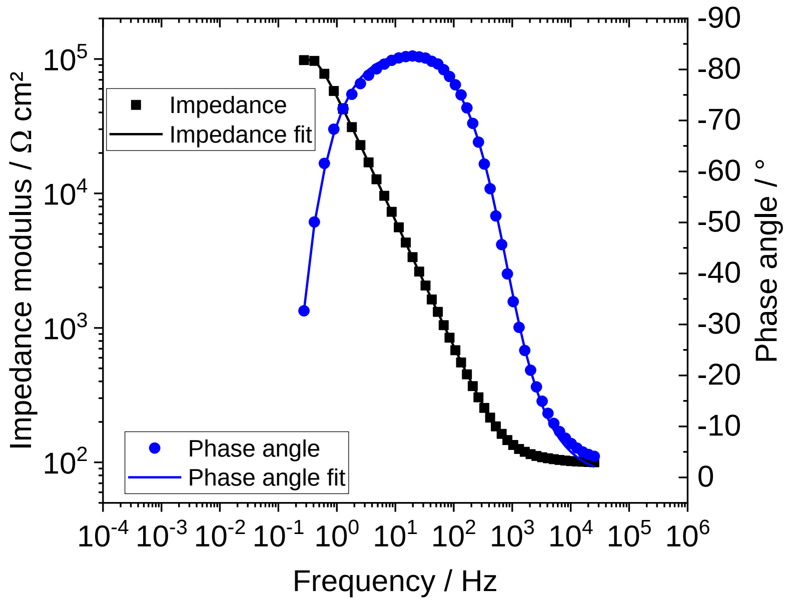

Figure S20. Electrochemical impedance spectrum (Bode plot) of a native oxide covered aluminum electrode recorded at open circuit potential immediately after immersion in Uralumina 150. Lines are best fits in the frequency range 25 kHz to 0.3 Hz to the equivalent circuit of pitting aluminum surfaces in **Figure 4b**.
